# Role of the calcium stimulation test in diagnosing medullary thyroid cancer: is it adequate to achieve a diagnosis in both sexes? An individual patient data meta-analysis

**DOI:** 10.1530/ETJ-24-0347

**Published:** 2025-05-29

**Authors:** Franz Sesti, Tiziana Feola, Pasquale Dolce, Valentina Guarnotta, Alessandro Veresani, Elia Guadagno, Filomena Bottiglieri, Maria Grazia Tarsitano, Andrea M Isidori, Annamaria Colao, Antongiulio Faggiano, Elisa Giannetta, on behalf of NIKE group

**Affiliations:** ^1^ Department of Experimental Medicine, Sapienza University of Rome, Rome, Italy; ^2^ Neuroendocrinology, Neuromed Institute, IRCCS, Pozzilli, Italy; ^3^ Department of Translational Medical Science, Federico II University of Naples, Naples, Italy; ^4^ Department of Health Promotion, Mother and Child Care, Internal Medicine and Medical Specialties, Section of Endocrinology, University of Palermo, Palermo, Italy; ^5^ Endocrinology Unit, Department of Internal Medicine and Medical Specialties (DiMI), University of Genoa, Genoa, Italy; ^6^ Department of Neurosciences, Reproductive Sciences and Dentistry, Gynecology Unit, Federico II University Hospital of Naples, Naples, Italy; ^7^ Endocrinology, Diabetology and Andrology Unit, Department of Clinical Medicine and Surgery, Federico II University of Naples, Naples, Italy; ^8^ Department of Medical and Surgical Science, University Magna Grecia, Catanzaro, Italy; ^9^ Centre for Rare Diseases (ENDO-ERN accredited), Policlinico Umberto I, Rome, Italy; ^10^ Endocrinology Unit, Department of Clinical and Molecular Medicine, Sant'Andrea Hospital, ENETS Center of Excellence, Sapienza University of Rome, Rome, Italy

**Keywords:** medullary thyroid cancer, calcitonin, calcium stimulation test, sex difference, meta-analysis

## Abstract

**Background:**

Early diagnosis of medullary thyroid cancer (MTC) when basal calcitonin (CT) levels are <100 pg/mL remains a clinical challenge. The calcium stimulation test is a unique tool for stimulating CT. However, standardized and sex-specific cutoff values are lacking.

Therefore, this study aimed to investigate whether the calcium stimulation test for CT is adequate for diagnosing MTC in both sexes and to identify sex-specific cutoff values.

**Methods:**

This was an individual patient data (IPD) meta-analysis. A literature search was performed using Scopus, PubMed, and Web of Science until September, 2024, to identify articles on the calcium stimulation test for diagnosing MTC.

**Results:**

A total of five studies involving 243 patients (148 females and 95 males) who underwent total thyroidectomy were included in this study. Before surgery, all patients underwent the calcium stimulation test with calcium gluconate (25 mg/kg) for CT assessed by chemiluminescence assay. In females, a global threshold of 162 pg/mL was identified, with a pooled sensitivity of 0.90 (95% confidence interval (95% CI): 0.79–0.97) and specificity of 0.66 (95% CI: 0.56–0.75). The pooled area under the curve (AUC) was 0.87 (95% CI: 0.76–0.97). In males, a global threshold of 562 pg/mL was identified, with a pooled sensitivity of 0.79 (95% CI: 0.60–0.92) and specificity of 0.89 (95% CI: 0.79–0.96). The pooled AUC was 0.94 (95% CI: 0.90–0.99).

**Conclusions:**

The calcium stimulation test for CT for the diagnosis of MTC showed better performance in males than in females, with a suggested cutoff value of 562 pg/mL in males.

**Significance statement:**

The management of indeterminate calcitonin (CT) values is still challenging in the early diagnosis of MTC, lacking general recommendations, which can help clinicians in these cases. This is the first IPD meta-analysis that underscores the sex-based disparity in the diagnostic accuracy of the calcium stimulation test for CT in suspected MTC cases, showing better performance in diagnosing MTC in male versus female patients, with a cutoff value of 562 pg/mL in male subjects. In the context of the limited literature, this paper provides added value for the clinical endocrine practitioner, suggesting the use of the calcium stimulation test in highly selected cases with indeterminate CT values (10–100 pg/mL) with a sex-oriented and personalized approach.

## Introduction

Medullary thyroid cancer (MTC) is a rare neuroendocrine tumor originating from parafollicular cells (C cells), with a prevalence of 0.21 per 100,000 people (1). MTC accounts for 3–5% of all thyroid cancers and up to 15% of all thyroid cancer-related deaths (2). It occurs sporadically in 75% of cases and is hereditary in 25% (3).

Calcitonin (CT), which is produced by thyroid C cells, is generally considered a highly sensitive and specific marker for MTC after excluding confounding factors (4). Indeed, many factors, such as individual factors such as age (elevated values in children under 3 years of age) and sex (higher values in males compared with females), drugs, smoking, and clinical conditions, may influence CT levels (5). Hypercalcemia, extra-thyroid neuroendocrine neoplasms, chronic renal failure, hepatic cirrhosis, chronic pulmonary disease, pseudohypoparathyroidism, and assay methodology can falsely increase CT values (6, 7, 8, 9, 10, 11, 12). Conversely, recent data did not support that autoimmune thyroiditis, hypergastrinemia related to autoimmune atrophic gastritis, or differentiated thyroid carcinoma can affect CT levels in patients with thyroid nodules, suggesting that in these conditions, hypercalcitoninemia should be submitted to the same diagnostic work-up in the suspicion of MTC (13, 14). However, not all scientific societies generally recommend routine assessment of CT values in patients with thyroid nodules. The American Thyroid Association, American Association of Clinical Endocrinologists, American College of Endocrinology, and Associazione Medici Endocrinologi guidelines do not recommend for or against routine CT testing, whereas the European Thyroid Association’s consensus-based guidelines recommend determination of CT levels in all patients with thyroid nodules (5, 15, 16). In addition, many studies have shown that a CT assay is helpful in early detection of MTC, enabling curative surgery and improved survival (17, 18, 19, 20). Basal serum CT levels above 100 pg/mL are highly suspicious for MTC, justifying a more aggressive approach, although lower values do not exclude MTC (5). When CT levels moderately increase, the calcium stimulation test is suggested to improve diagnostic performance in different clinical settings (for the timing of thyroidectomy in children who have inherited a mutated Ret allele, for the evaluation of patients for persistent or recurrent MTC following thyroidectomy, and for detecting MTC in patients with nodular goiters) (5). Moreover, this test could be useful in the differential diagnosis between CT-secreting neuroendocrine neoplasms and MTC because serum CT levels do not increase in response to calcium stimulation in patients with the various neuroendocrine neoplasms (21).

Currently, intravenous calcium administration is considered a unique option for stimulating CT because of the unavailability of pentagastrin in the last years; it has a low cost with a better safety profile because it is associated with a low number and intensity of side effects compared with pentagastrin (22). Calcium is a potent secretagogue stimulating CT release, with peak CT values observed 2 min after stimulation and higher values observed in males than in females (23, 24). However, no standardized basal and stimulated CT cutoff values have been reported because of differences in interlaboratory and interassay CT measurements (21).

Therefore, this study aimed to investigate whether the calcium stimulation test for CT is adequate for the diagnosis of MTC in both sexes and to identify sex-specific cutoff values. We performed an individual patient data (IPD) systematic review and meta-analysis of the available published studies to achieve this aim.

## Materials and methods

This IPD systematic review and meta-analysis was performed following a rigorous protocol in accordance with the Reporting Items for Systematic Reviews and Meta-Analyses of Individual Participant Data (PRISMA-IPD) guidelines (25). This study was not registered with PROSPERO.

### Search strategy

The primary search was conducted using Scopus, PubMed, and Web of Science databases until September 30, 2023, to identify articles on the calcium stimulation test for CT. In addition, we searched the reference lists of the retrieved articles for relevant articles. The search was restricted to articles published in English. The electronic databases were searched using the following keywords: (calcium gluconate stimulating test AND calcitonin) OR (calcium stimulating test AND calcitonin) OR (calcium gluconate stimulating test AND CT) OR (calcium stimulating test AND CT). A final update of the search was conducted in September, 2024; no more studies were included.

### Study selection

**Figure 1:**
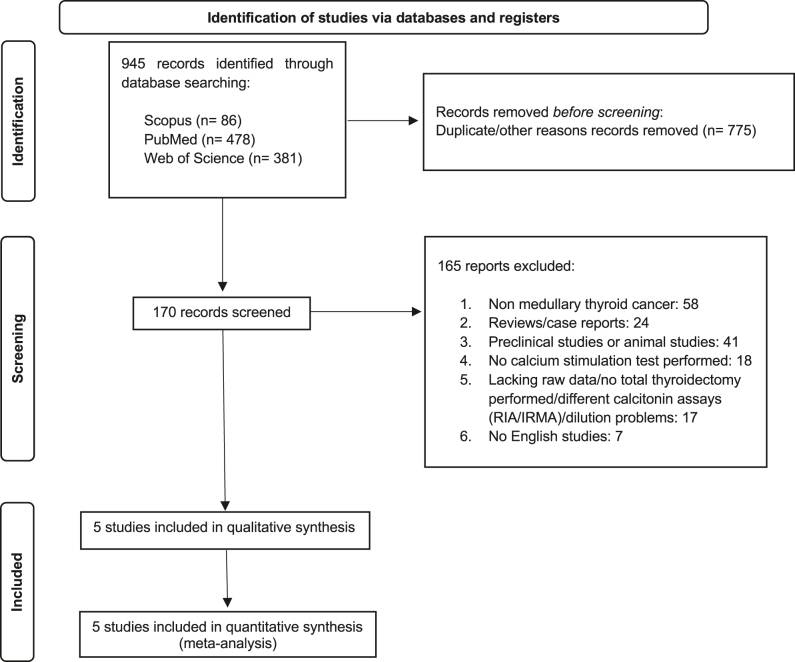
Flow diagram for study selection (search until September 2024). RIA, radioimmunoassay; IRMA, immunoradiometric assay

All studies that met the following eligibility criteria were included: i) prospective and retrospective observational cohort studies, ii) studies reporting individual data for each patient, iii) calcium stimulation test performed with calcium gluconate 25 mg/kg (elemental calcium 2.3 mg/kg) for patients operated for thyroid nodules with biochemical or cytological suspicion of MTC and/or tracheal compression, iv) basal and stimulated CT levels assessed by chemiluminescence assay (CLIA), and v) patients with a negative germline *RET* gene mutation analysis. Reviews, animal studies, case reports, and non-original articles were excluded. All identified titles and abstracts were independently screened by four reviewers (F S, E G, V G, and A V), and potentially eligible articles were evaluated. Corresponding authors were contacted in case the full texts or IPD were unavailable. A high level of interobserver agreement was observed (95%). Any disagreement between the reviewers was resolved by reaching a consensus through discussion. Figure 1 shows the study selection process.

### Data extraction and quality assessment

Data were independently extracted by five reviewers (F S, E G, T F, V G, and A V), including study design, study population, age, sex, *RET* gene mutation status, dose and time points of the calcium stimulation test, assay of CT measurement, basal and peak of stimulated CT of each patient, histological report (benign, C-cell hyperplasia (CCH), or MTC), and basal and stimulated CT cutoff values according to sex. *RET* mutation carriers were excluded to avoid possible interference exerted by diffuse CCH in the determination of the cutoff values. Overlapping data in articles that partially involved the same patients were excluded to avoid duplication. Table 1 shows the details of the included studies. Quality control checks on extracted data were performed by another investigator (T F). The risk of bias was independently assessed for all studies by two reviewers (T F and F S) involved in study selection and extraction using the Quality Assessment of Diagnostic Accuracy Study-2 (QUADAS-2) tool (26). Each item was evaluated as having a low, unclear, or high risk of bias (Table 2). Any disagreement was resolved by a third reviewer (E G).

**Table 1:** Characteristics of the studies selected for analysis.

Study	Study design	Selected condition	Ca stimulating test			TD patients			Age range (years)	Patients included *		
			CAG dose, mg/Kg	Elemental Ca, mg/Kg	Time, minutes	Total	Male	Female		Total	Male	Female
Colombo *et al.* (31)	PMOC	TOC	25	2.3	0, 2, 5, 15	40	15	25	26–76	36	15	11
Faggiano *et al.* (28)	RMLC	TOC	25	2.3	0, 2, 5, 10	90	40	50	18–20	30	7	23
Fugazzola *et al.* (32)	PMLC	TOC	25	2.3	0, 2, 5, 10	54	21	33	35–82	54	21	33
Mian *et al.* (23)	PMOC	TOC	25	2.3	0, 2, 5, 10	91	42	49	8–81	82	38	44
Rosario *et al.* (33)	PMOC	TOC		2.5	0, 2, 5, 10	41	14	27	16–78	41	14	27

* After exclusion of RET mutation carriers or subjects with serum CT assessed by immunoradiometric assay (IRMA).

PMOC, prospective, monocenter; PMLC, prospective, multicenter; RMLC, retrospective, multicenter; CAG, calcium gluconate; TOC, thyroid-operated cases; TD, thyroidectomized.

**Table 2:** Meta-analysis of the calcium stimulation test for CT in females for the diagnosis of MTC.

Study	Threshold	TP	FN	FP	TN	Sensitivity	Specificity
Colombo *et al.* (31)	162	7	0	2	12	1 (0.59–1)	0.86 (0.57–0.98)
Faggiano *et al.* (28)	162	10	2	3	8	0.83 (0.52–0.98)	0.73 (0.39–0.94)
Fugazzola *et al.* (32)	162	18	1	13	1	0.95 (0.74–1)	0.07 (0–0.34)
Mian *et al.* (23)	162	8	2	7	27	0.8 (0.44–0.97)	0.79 (0.62–0.91)
Rosario *et al.* (33)	162	3	0	8	16	1 (0.29–1)	0.67 (0.45–0.84)
Global	162	46	5	33	64	0.90 (0.79–0.97)	0.66 (0.56–0.75)

TP, true positives; FN, false negatives; FP, false positives; TN, true negatives.

### Data synthesis and statistical analysis

Receiver operating characteristic (ROC) curve analysis was performed using data integrated from each study. This analysis aimed to identify a unique global optimal cutoff value that was applied across all primary studies. A unique global optimal cutoff prevents a lack of clinical relevance and potential biases in test performance estimates, which could arise from employing different cutoff values, although optimal in each specific study. Subsequently, the sensitivity and specificity were estimated for each study.

Furthermore, both fixed-effect and random-effect models were used to assess the accuracy of the calcium stimulation test for CT, accounting for patient clustering within studies. Pooled estimates of the area under the curve (AUC) were calculated, and all results were presented in forest plots. The heterogeneity of AUC across studies was evaluated using the *I*^2^ statistic and Cochran’s Q test, categorizing heterogeneity into low (*I*^2^ < 25%), moderate (*I*^2^ = 25–50%), and high (*I*^2^ > 50%).

All statistical analyses were performed using the R statistical software. IPD meta-analysis for diagnostic test accuracy was performed using IPDmada, a web-based R Shiny application (27). Estimates were reported along with their 95% confidence intervals (95% CI).

## Results

### Study selection

A total of 945 potentially relevant studies were identified. Among them, 775 were excluded before screening (duplicates or other reasons), and 170 were evaluated by title and abstract. Of the 170 studies, 165 were excluded due to the following reasons: not written in English, reviews or case reports, preclinical or animal studies, not MTC, calcium stimulation test not performed, missing IPD, total thyroidectomy not performed, different CT assays (immunoradiometric assay), and dilution issues. Finally, five eligible studies were included in the qualitative and quantitative analyses. Figure 1 shows the study selection process. Only one study (28) included more than one CT assay. In this case, only CT data assessed by CLIA were analyzed.

### Study characteristics

Table 1 shows the main characteristics of the included studies. All studies, except one (28), were prospective studies involving 243 patients (95 males and 148 females) aged 8–82 years who underwent total thyroidectomy for thyroid nodules suspicious for MTC, tracheal compression, or cytological suspicion of MTC. All patients underwent the calcium stimulation test before surgery with calcium gluconate (25 mg/kg) or elemental calcium (2.3–2.5 mg/kg) for CT assessed by CLIA at the following time points: 0, 2, 5, 10, and 15 min.

### Diagnostic accuracy of the calcium stimulation test for CT in females

**Figure 2:**
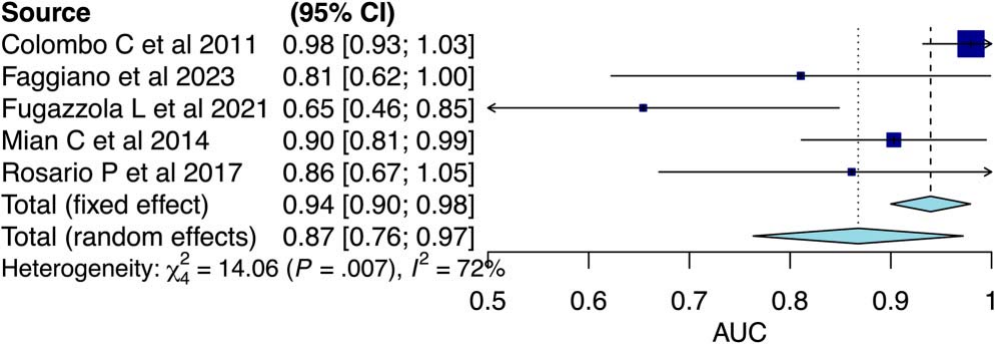
Meta-analysis of AUC in females.

**Figure 3:**
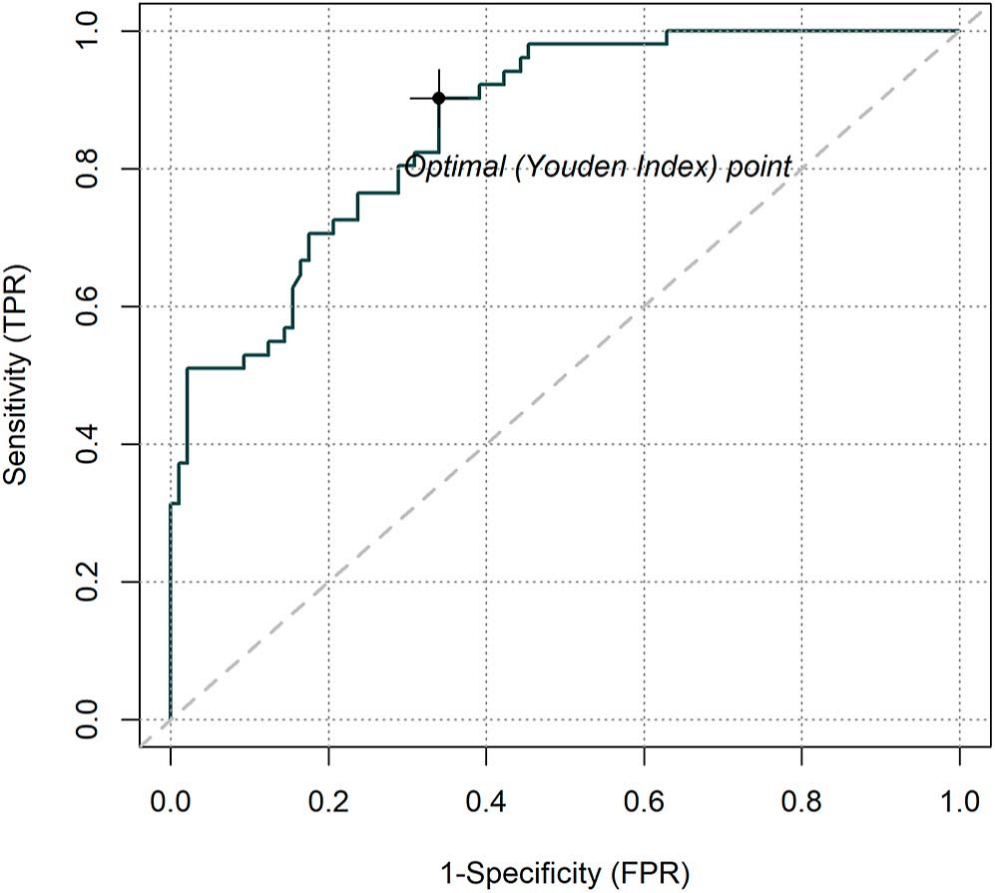
Summary ROC curve for the calcium stimulation test for CT in females. AUC = 0.86; optimal cutoff = 162 pg/mL.

Analysis was performed according to sex. The results showed a global threshold of 162 pg/mL in females with a pooled sensitivity of 0.90 (95% CI: 0.79–0.97) and specificity of 0.66 (95% CI: 0.56–0.75) (Table 2). The pooled AUC was 0.87 (95% CI: 0.76–0.97) (Fig. 2). Figure 3 shows the ROC curve.

### Diagnostic accuracy of the calcium stimulation test for CT in males

**Figure 4:**
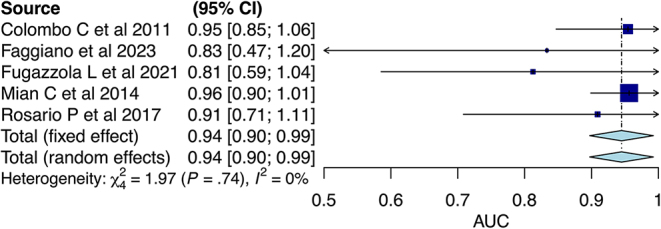
Meta-analysis of AUC in males.

**Figure 5:**
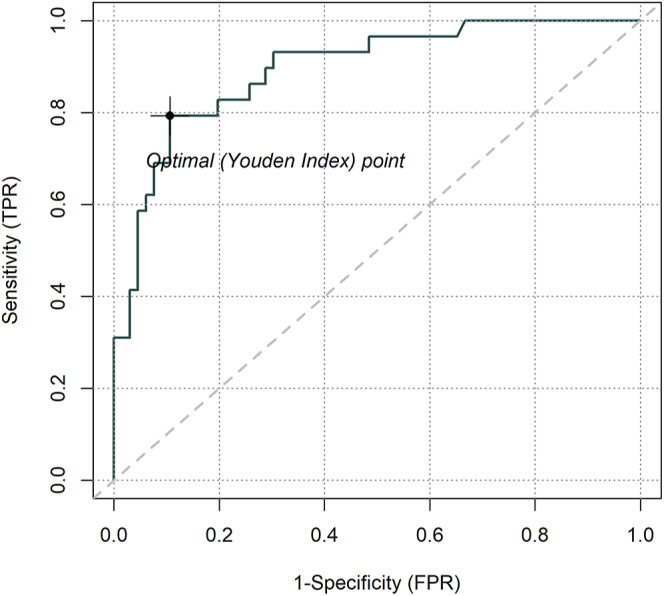
Summary ROC curve for the calcium stimulation test for CT in males. AUC = 0.90; optimal cutoff = 562 pg/mL.

The results showed a global threshold of 562 pg/mL in males with a pooled sensitivity of 0.79 (95% CI: 0.60–0.92) and specificity of 0.89 (95% CI: 0.79–0.96) (Table 3). The pooled AUC was 0.94 (95% CI: 0.90–0.99) (Fig. 4). Figure 5 shows the ROC curve.

**Table 3:** Meta-analysis of the calcium stimulation test for CT in males for the diagnosis of MTC.

Study	Threshold	TP	FN	FP	TN	Sensitivity	Specificity
Colombo *et al.* (31)	562	4	0	2	9	1 (0.4–1)	0.82 (0.48–0.98)
Faggiano *et al.* (28)	562	1	2	0	4	0.33 (0.01–0.91)	1 (0.4–1)
Fugazzola *et al.* (32)	562	6	2	1	12	0.75 (0.35–0.97)	0.92 (0.64–1)
Mian *et al.* (23)	562	10	1	3	24	0.91 (0.59–1)	0.89 (0.71–0.98)
Rosario *et al.* (33)	562	2	1	1	10	0.67 (0.09–0.99)	0.91 (0.59–1)
Global	562	23	6	7	59	0.79 (0.60–0.92)	0.89 (0.79–0.96)

TP, true positives; FN, false negatives; FP, false positives; TN, true negatives.

### Risk of bias assessment, publication bias, and heterogeneity

The QUADAS-2 scores indicated that the quality of the included studies was high (Table 4). Funnel plot analysis could not be appropriately performed because of the limited number of studies. No significant heterogeneity was observed in males (*I*^2^ = 0%). However, it was high in females (*I*^2^ = 72%).

**Table 4:** Risk of bias summary using the QUADAS-2 tool.

Study	Risk of bias				Applicability concerns		
	Patients selection	Index test	Reference standard	Flow and timing	Patients selection	Index test	Reference standard
Colombo *et al.* (31)	⊖⊕	⊖	⊖	⊖	⊖	⊖	⊖
Faggiano *et al.* (28)	⊕	⊖	⊖	⊖	⊖	⊖	⊖
Fugazzola *et al.* (32)	⊖	⊖	⊖	⊖	⊖	⊖	⊖
Mian *et al.* (23)	⊖	⊖	⊖	⊖	⊖	⊖	⊖
Rosario *et al.* (33)	⊖	⊖	⊖	⊖	⊖	⊖	⊖

⊖ indicates ‘low risk’; ⊕ indicates ‘high risk’.

## Discussion

Early diagnosis of MTC when basal CT levels are indeterminate between 10 and 100 pg/mL is challenging in clinical practice, considering that ultrasound features and cytological results are often inconclusive and clear recommendations by the international societies are lacking (5, 29). Therefore, the calcium stimulation test can enhance accuracy in early diagnosis of MTC before the occurrence of lymph node metastases (up to 43% in micro-MTC) (30) in patients with thyroid nodules and indeterminate values of basal CT, thereby optimizing surgical procedures and improving survival. Previous studies have proposed different cutoff values according to sex, demonstrating higher values in males than in females; however, these studies have shown high variability and no agreement on the best cutoff values (23, 28, 31, 32, 33, 34, 35). The different basal and stimulated CT values between males and females are probably due to a higher C cell density (more than twice) and greater prevalence of CCH in normal male adults than in female ones (36). To the best of our knowledge, this is the first meta-analysis that explored sex differences in the diagnostic accuracy of the calcium stimulation test for CT for the diagnosis of MTC, showing a high accuracy of the test with a better performance in males than females. In males, stimulated CT showed an excellent pooled AUC (0.94) without heterogeneity among the studies, indicating that this test is a helpful tool in the diagnostic work-up of MTC when basal CT levels are inconclusive. Conversely, in females, stimulated CT showed a good pooled AUC (0.87). However, the analysis was limited by the high heterogeneity among the included studies. Moreover, this meta-analysis confirmed the data in the literature on the need for sex-specific cutoff values for early diagnosis of MTC with good accuracy, providing for the first time a global threshold (stimulated CT threshold of 562 pg/mL in males and 162 pg/mL in females).

Despite the strong diagnostic performance of the proposed cutoffs, it is important to acknowledge their variability in pooled sensitivity and specificity, particularly in female patients, as reflected by a pooled specificity of 0.66. This highlights the need for careful interpretation of stimulated CT values in females and the necessity of integrating additional diagnostic tools to refine risk stratification and avoid unnecessary procedures.

Identifying sex-specific thresholds for the calcium stimulation test contributes to guiding clinical decision-making, especially in cases where traditional diagnostic modalities yield equivocal results, causing potential diagnostic delays or missed treatments. Neither single nor the association of ultrasound features is specific for MTC (37). Several scientific societies proposed ultrasound risk stratification systems that represent the main tool for diagnosing the nodules, but they have a suboptimal accuracy in detecting MTC, considering that MTC presentation according to the main ultrasound risk stratification systems is classified in a high-risk/suspicion category in just over half of cases (37, 38). Similarly, cytological examination of fine-needle aspiration (FNA) specimens is the main procedure for detecting papillary thyroid carcinoma, but it is able to detect approximately one-half of MTC lesions (39, 40, 41). Therefore, according to the American Thyroid Association Guidelines, measuring CT in washout fluid of FNA and immunohistochemistry staining of the FNA sample for CT are recommended tools to improve MTC diagnosis and overcome the limits of ultrasound and cytology (5).

Sex-specific thresholds for the calcium stimulation test can be leveraged to tailor diagnostic algorithms and treatment strategies to individual patient profiles, thereby optimizing therapeutic efficacy and patient outcomes. Further prospective and multicentric studies are needed to better define the role of the calcium stimulation test in detecting MTC, particularly in female subjects; it is essential to evaluate multiple aspects by integrating all the current tools to improve the diagnostic work-up of MTC with a sex-oriented and personalized approach.

### Strengths

The strengths of this study lie in its design, application of IPD systematic review and meta-analysis, adherence to rigorous methodology in accordance with the PRISMA-IPD guidelines, and ensuring transparency and reproducibility. In addition, a comprehensive search was performed with meticulous study selection and quality assessment using the QUADAS-2 tool. Moreover, *RET*-positive patients were excluded to avoid sources of heterogeneity. The test protocol was sufficiently homogeneous among the studies (calcium gluconate dose, time points, and CT assay).

### Limitations

Although this meta-analysis expands the knowledge about the calcium stimulation test for CT for the diagnosis of MTC, it has some limitations. Few studies met the inclusion criteria, with heterogeneous study populations and laboratories, preventing performing subgroup analysis (for example, for age groups to define children-specific cutoffs) or meta-regression and exploring publication bias. Finally, the underlying pathophysiological mechanisms driving sex-specific differences in MTC remain unclear. A role of sex hormones cannot be excluded and should be better investigated.

### Challenges and advances

The diagnostic evaluation of patients with suspected MTC is complex and involves multiple steps. The process begins with clinical assessment, including family history and individual risk factors, followed by thyroid function tests, ultrasound risk stratification, measurement of serum CT levels, FNA with CT measurement in the washout liquid, and imaging studies, such as total-body computed tomography and magnetic resonance . Each diagnostic approach has advantages and limitations, and understanding these aspects is crucial for detecting MTC at an early stage. Early detection significantly increases the likelihood of biochemical cure and improves patient prognosis.

Basal CT is a highly sensitive and specific biomarker for MTC (42). However, routine CT screening for thyroid nodules remains controversial due to: i) the low prevalence of MTC (≈0.32%), which reduces the positive predictive value of the test; ii) cost-effectiveness concerns, as widespread CT screening may not be financially justifiable; iii) overestimation risks, as studies lack adequate reference standards for patients with negative basal CT levels; and iv) pre-analytical and analytical variability, with basal CT levels influenced by physiological or pathological conditions, heterophilic antibodies, and macrocalcitoninemia (43).

In cases of borderline basal CT values and high clinical suspicion of MTC, a stimulation test may improve specificity with minimal impact on sensitivity (42). However, patients with CT-negative thyroid nodules or stimulated CT responses below diagnostic thresholds require long-term follow-up with integrated clinical, radiological, and biochemical monitoring.

Recently, procalcitonin (Pro-CT) has been proposed as an adjunctive test for detecting MTC, particularly in patients with slightly elevated CT levels (43). Pro-CT offers several advantages: i) greater stability and a longer half-life than CT; ii) good diagnostic accuracy in distinguishing MTC from benign conditions; and iii) potential prognostic value, correlating with tumor size and lymph node involvement. However, further research is needed to establish internationally accepted thresholds before Pro-CT can be routinely implemented in clinical practice (43).

Advances in genetic and molecular profiling have improved our understanding of MTC pathogenesis. RET mutations play a central role in hereditary MTC and occur in approximately 50% of sporadic cases, influencing disease progression. RAS mutations, reported in a subset of sporadic MTCs, and non-coding RNA alterations may further impact tumor behavior. Molecular profiling can aid in risk stratification, prognosis, and targeted therapy selection. An integrated diagnostic approach, combining genetic, molecular, and artificial intelligence-based analyses, could revolutionize MTC diagnosis and personalized treatment in the era of precision medicine (44).

## Conclusion

This meta-analysis underscores the sex-based disparity in the diagnostic accuracy of the calcium stimulation test for CT in suspected MTC cases. Although the test holds promise for male subjects, with a suggested cutoff value of 562 pg/mL, further investigation is needed to refine its utility in females. Caution should be exercised when interpreting test results, and complementary diagnostic modalities, including ultrasound risk features, serum CT levels, cytology, immunohistochemistry, and CT measurement in washout fluid of FNA, should be considered to ensure accurate diagnosis and appropriate clinical management. While calcitonin remains the cornerstone of MTC diagnosis, emerging biomarkers such as procalcitonin and advanced genetic/molecular profiling may further refine risk assessment and management strategies, ultimately improving patient outcomes.

## Declaration of interest

AMI has been a consultant for Novartis, Takeda, Recordati, and Sandoz companies and has received unconditional research grants from Shire, IPSEN, and Pfizer. All the other authors have no conflicts of interest.

## Funding

This research was supported by co-funding of the European Union – Next Generation EU, Mission 4 Component 2 Investment 1.5, project Rome Technopole – code ECS 00000024 (CUP: B83C22002820006).

## Author contribution statement

F S, E G, and A F conceived and designed the study. F S, E G, T F, V G, and A V collected the data and co-wrote the manuscript. P D conceived the statistical analysis. All authors contributed to the revision of the manuscript. All authors have read and approved the final version of the manuscript.

## Data availability

Some or all datasets generated during and/or analyzed during the current study are available from the corresponding author upon request.
